# Integrating homeless persons with mental health conditions back into low resource communities: A cross-sectional study

**DOI:** 10.1371/journal.pmen.0000510

**Published:** 2026-03-20

**Authors:** Alex Ansah Owusu, Frank Baning, Leveana Gyimah, Dansoa Nuamah, Julius Xatse, Akua Owusuaa Gyapong, Royal Konlaan, Daniel Kudzo Fiawotror, Bernard Mortotsi, Joseph Bediako Asare

**Affiliations:** 1 Department of Public Health, Pantang Hospital, Pantang, Ghana; 2 Mental Health Authority of Ghana, Accra, Ghana; 3 WHO Country office, Accra, Ghana; 4 Department of Psychiatry, Pantang Hospital, Pantang, Ghana; 5 Department of Psychiatry, Ankaful Psychiatric Hospital, Ankaful, Ghana; 6 Department of Community Psychiatry, Pantang Hospital, Pantang, Ghana; 7 Department of Social Welfare, Pantang Hospital, Pantang, Ghana; 8 Department of Finance, Accra Psychiatric Hospital, Accra, Ghana; 9 Psychiatrist of Ghana, Accra, Ghana; National University of Singapore, SINGAPORE

## Abstract

Homeless mentally ill persons remain a visible public health problem across Ghana. This study describes a reintegration intervention that identifies homeless persons with mental health conditions from the streets, provides comprehensive physical and mental healthcare, traces and reunites them to their families, links them to livelihoods and community mental-health services, and conducts routine follow-up calls. Data for this study were obtained from records review. Since the project began in 2016, 81 beneficiaries (53 females, 28 males) had been enrolled by 31 December 2024, with street exposure ranging from 11 days to 17 years; 28.4% had a history of psychoactive substance use, 66.7% had prior psychiatric care, and 91.4% were diagnosed with schizophrenia. At data compilation 29.6% remained on admission and 60.5% (49 people) had been repatriated, most after 1–3 months. Total expenditure for all 81 beneficiaries was GHS 2,835,365.30 (USD 190,933.69); spending on the 49 repatriated beneficiaries totalled GHS 1,635,620.00 (USD 110,143), averaging GHS 33,380 (USD 2,248) per person and ranging from GHS 14,019.60 to GHS 54,743.20. Among repatriated beneficiaries, 11.1% were engaged in economic activities, 55.6% assisted with household chores but were not employed, and 8.9% relapsed. With sustained stakeholder support and active collaboration, the intervention could serve as a scalable model for addressing homelessness among people with mental illness.

## Introduction

Homeless mentally ill persons continue to populate the streets of Ghana’s capital. The situation is no different all over Ghana. On their own, individuals with mental illnesses are usually unable to access basic needs and are predisposed to experiencing a deterioration in their overall well-being [1]. The mentally unwell are at an increased risk of communicable and non-communicable diseases as well as injuries, whether deliberate or not. Homelessness and comorbidity of conditions serve a negative index on prognosis for these individuals and thus require attention owing to their humanitarian and public health implications [2]. A case in point is the fact that whilst unwell, they are to a large extent unable to adhere to outlined public health interventions like the Covid-19 protocols, despite being as susceptible as the general populace.

In Ghana, over the years until the year 2000, whenever there was an international event which involved international foreign dignitaries, a task force consisting of the Police, the Department of Social Welfare and the Ministry of Health, specifically Accra Psychiatric Hospital, came together to arrest vagrants living on the streets of Accra in a day. The victims were all kept at a large compound at the Accra Psychiatric hospital and a team of specialists screened them to retain the mentally ill and the rest were taken over by the department of social work, who tried to link them with their families, while some who were arrested by their appearance but were stable were discharged. Unfortunately, these vagrants returned to the streets within a month, thus questioning the usefulness of the exercise. Appropriate data were not kept on these interventions.

Albeit existing legislation governing the provision of mental health services in Ghana [3], a myriad of stop-gaps still militate against its effectiveness. Some of these factors are:

- Lack of implementation and funding of established policies that cater for the mentally ill [4,5].
- Poverty which is negatively impacting the accessibility of mental health services [6].
- Poor mental health-seeking behaviour due to the stigma associated with mental illness and the beliefs associated with the causation of mental illness [7].

### Poor community-based support for persons with mental illness [4]

In a systematic review conducted by Fazel et al. in 2008 pertaining to homeless people in Western countries, homelessness was positively related to alcohol, drug dependence, psychotic and personality disorders, with prevalence estimates of 8.5%-58.1%, 4.7%-54.2%, 2.8%-42.3% and 2.2%- 71.0% respectively [8]. A few years further down the line, in 2011 in Denmark, Nielsen et al. found prevalence estimates of about 61.1% and 45% for psychiatric disorders and substance abuse disorders, respectively, amongst homeless sheltered persons [9]. This trend of establishing a high prevalence of mental illness amongst homeless populations was replicated by another study in 2015 [10] as well as much earlier studies [11]. More recently, a meta-analysis by Tinland et al. reported a prevalence rate, regarding mental illness, of 42% amongst homeless populations [12]. The picture in Canada, Australia, Japan and Hong Kong is not any different with high prevalence rates reported by studies conducted [13–16].

Leaving no one behind in our quest to achieve the Sustainable Development Goals means ensuring quality health, ending poverty, and ensuring food security for homeless mentally ill people too [17]. In an effort to address this public health problem, the Pantang Hospital started the “Set the Captives Free” project (STCF). This project is aimed at the management, rehabilitation, reintegration into communities, enhancement of productivity and relapse prevention for persons with mental health disorders wandering the region’s streets- a less-capital-intensive intervention is employed by the American Association of Community Psychiatrists owing to the overwhelming mental health needs of street populations in the United States [18]. A similar study from Ethiopia in 2014 by Fekadu et al. found prevalence values of 41% for psychosis and 60% for some form of alcohol use. They also found 10% of those experiencing psychosis had had treatment in the past [19]. An even more similar intervention in the neighbouring country of Nigeria, published in 2016 by Nwaopara, Abasiubong and Umoh had interesting results. 22.9% of beneficiaries had substance-related problems, with 37% being diagnosed with schizophrenia. Noteworthy also was the high prevalence of unemployment, 89.9%, and being single, 82.2%, in the study population [20]. Collectively, these figures highlight the need for attention for homeless populations [8,9,19–21].

Finances play an important role in the management of mental disorders. Hwang et al., in 2011, assessing costs and length of stay among homeless patients admitted to medical, surgical and mental health services, found the cost of admission of homeless people for psychiatric care to be approximately $1058 more than that of housed-patient admissions, irrespective of duration of stay [22]. According to this research conducted in Philadelphia by Dr. Stephen Poulin and his associates at the University of Pennsylvania, the average annual cost of public services for mentally ill homeless people is $22,372. The researchers noted that “the study did not include the costs connected with the police, courts, emergency medical services, and health care not associated with behavioural health”. Hence, the actual cost per person was substantially more than $22,372. The article noted that good psychiatric services might be offered for that kind of money if the care was well-managed. Hence, it would not only be compassionate but also financially prudent to ensure that mentally ill homeless individuals received appropriate psychiatric treatment [23].

The study presents the methods and preliminary findings from an intervention that seeks to provide a reproducible, sustainable model for tackling the problem of mentally ill persons roaming the streets of major cities. It also describes the major challenges associated with this approach.

## Method

### Study design

A descriptive cross-sectional study was conducted using secondary data.

### Study setting

#### Greater accra region.

The Greater Accra Region has the smallest area of Ghana’s 16 administrative regions, occupying a total land surface of 3,245 square kilometres. This is 1.4 per cent of the total land area of Ghana. It is the most populated region, with a population of 5,455,692 in 2021, accounting for 17.7 per cent of Ghana’s total population [24].

The Greater Accra region is the most urbanised region in the country, with 87.4% of its total population living in urban centres. The capital city of the Greater Accra Region is Accra, which is at the same time the capital city of Ghana [24].

The Greater Accra Region is bordered on the north by the Eastern Region, on the east by the Volta Region, on the south by the Gulf of Guinea, and on the west by the Central Region.

1.3% of the inhabitants of the Greater Accra Region are immigrants from outside Ghana.

The largest portion of the population of Accra is Akan, at 39.8% of the population. The next largest group is Ga-Dangme at 30.7% of the population. After this 18% of the population is Ewe.

The religious affiliations of the people of the Greater Accra region are below:

Christian – 77.8%, Muslim –16.2%, Other Religions – 4.6% and Traditional – 1.4% [23].

#### Pantang hospital.

Pantang Hospital is the largest of the three psychiatric hospitals in Ghana. The facility was commissioned in 1975. Pantang Hospital was meant to be a tertiary training centre of excellence in research and mental health care for the West African Sub-region and beyond. It is 25 kilometres from Accra Central. The hospital was originally planned to be a Pan-African Mental Health Village [25].

The hospital currently operates a 200-bed capacity facility catering for in-patient and out-patient psychiatric and general medical services. Because the hospital provides other services beyond psychiatric care, the facility is better placed to render integrated healthcare to clients.

The hospital has an average annual client attendance of 43,883. The majority of the hospital’s clientele are Ghanaians, with a few hailing from other West African countries like Togo, Benin, Burkina Faso and the Ivory Coast.

### Study participants and sampling

The study reviewed the health records of all project beneficiaries; these are persons with mental illness who lived on the streets before benefitting from this intervention.

### Data collection

Data compilation forms were used to collect data from the health records of beneficiaries (as attached in S1 Text) and the database for reintegrated beneficiary follow-up calls (as attached in S2 Text). Data extraction was from 6^th^ to 13^th^ of January 2025. Data were deidentified by the first author before the analysis hence, none of the other authors had access to information that could identify the project beneficiaries whose data were included in this study.

### Data collection techniques and tools

Records review was used to collect data with the aid of data compilation forms. The data compilation forms collected data on beneficiary demographics, pick-up and repatriation data, diagnosis and treatment and post-repatriation data.

Community psychiatric nurses of Pantang Hospital were trained to collect data from both project beneficiary folders and electronic health records. Data were also collected from the patient follow-up call records. These are notebooks at the community psychiatric unit and social welfare unit in which reports generated after calling discharged hospital clients, which include project beneficiaries, are documented.

### Inclusion criteria

Data on all project beneficiaries who were recruited before the end of the year 2024 were included. There were no exclusion criteria.

### Quality control

To guarantee the quality of the study outcome, certain measures were put in place throughout the data collection process. These measures were as follows.

The data compilation forms were scrutinised for mistakes and completeness.

Entries that were unclear or had a lot of missing information were not included in the analysis; missing data were reported as “unknown” or “could not be reached”

Entered data were cross-checked to reduce entry errors and to make the data more reliable.

### Data management and analysis

Data were entered into Microsoft Excel (as attached in S1, S2 and S3 Data) and analysed using STATA version 15. Mean and standard deviation were computed for age and other continuous variables. Frequencies and percentages were presented with the aid of tables. The cost of managing beneficiaries was computed from the patient bills generated for services used, the cost involved in picking up beneficiaries from the streets and the cost of transporting beneficiaries to their families. The mean cost of managing all project beneficiaries was calculated.

### Ethical considerations

Ethical approval was obtained from the Ghana Health Service Ethics Review Committee (GHS-ERC Number: **GHS-ERC:003/08/23**). Approval was also obtained from the Mental Health Authority and the hospital director for Pantang Hospital.

### Intervention flow

#### Inputs.

This project brings on board the services of a multidisciplinary team made up of community psychiatric nurses (CPN), psychiatrists, clinical psychologists, occupational therapists, pharmacists, social welfare personnel, general medical practitioners, dental surgeons, drivers, security personnel and information technology officers (IT) of the hospital.

Currently, the project is mainly funded by corporate institutions, the Mental Health Authority (MHA), churches, individuals and the hospital.

#### Process.

##### Pre-pick up activities and surveillance:

Details of how one homeless mentally ill person living on the street is selected over another are discussed in a different manuscript, but priority is given to younger persons. The project also prioritises persons whose state and or location poses a risk to their lives or that of others. For example, mentally ill persons on busier streets are prioritised over those on less busy streets. Also, those involved in road traffic injuries or with other visible medical conditions are given priority.

The client is identified by the CPN, after which the intention to make the client a beneficiary of the project is communicated to the police within the district. A formal request is also made for an involuntary admission court order for medical and psychiatric care.

The next activity is getting evidence of the client’s routine activities on the street (videos, pictures of daily activities).

##### Activities during client pick-up:

The pick-up team is transported to the pick-up location, directed by the community psychiatric nurses. The IT professional takes photographs and videos of the process. The client may be sedated where necessary and then transported to the hospital. Even though sedation is hardly used, it is employed as a last resort in situations where the prospective beneficiary is aggressive and poses a threat to lives and property in the community.

##### Activities after the client arrives at the hospital:

The client is sent to the Psychiatric OPD and goes through the routine consultation and admission processes. The client is then enrolled on the national health insurance scheme (NHIS) by the Social Welfare team. NHIS covers some of the physical care services, but not the mental health services. The IT team creates a folder for the client where monthly reports and pictures of the client are stored.

##### Care in the Hospital:

The client receives comprehensive health care services. These include, but are not limited to, a psychiatric consultation, psychotherapy, occupational therapy(OT), a physician consultation and a dental consultation.

During OT, beneficiaries are engaged through individual therapy sessions and group therapy sessions, depending on their needs, presentation, and cognitive abilities. OT is used to improve beneficiaries’ communication skills through guided group discussions that strengthen listening, turn-taking, and reciprocal conversation. It also introduces clients to a variety of group leisure activities such as art, craft, music and games to enhance cooperation, enjoyment, and rule-following. Memory games and cognitive activities are used to help clients strengthen recall, sequencing, and retention of information. OT is used to provide exposure to pre-vocational group tasks such as cooperative work, simple assembly and chore routines to build work habits and functional task behaviours. Through personalised support and therapeutic routines, the program empowers clients to build independence, emotional stability, and practical skills essential for community reintegration and long-term wellbeing.

In instances where the client requires specialist care not offered in Pantang Hospital, the client is sent to a referral facility for the needed service.

CPN and Social Welfare officers trace and contact relatives of the client. Family tracing is done using a number of approaches listed below:

Beneficiaries are interviewed by CPNs and Social welfare officers to obtain information on their names, places of residence before living on the streets, as well as names and phone numbers of family members or friends. When beneficiaries give consistent information over two or more interviews, the information is deemed reliable for family tracing. If phone numbers are provided, they are called. When this fails, the facility CPNs and social welfare officers share the information with their colleagues in the districts of residence provided by the beneficiary. Via this approach, the project rides on existing national structures to reduce the burden on hospital resources. Family or friends identified by the district team are invited to the hospital. In instances where the approaches described do not work, the hospital CPNs and social workers allow the beneficiaries to lead them to their houses using a hospital van. The project has also used Facebook and the NHIS database to obtain relevant family details.

Identified relatives are educated on the client’s condition and the role of the family in the recovery process. When the client is due for discharge, the reintegration process follows. The project has also considered the need for beneficiaries to review their pick-up pictures and videos to help them appreciate the need to continue to take their medication and comply with treatment.

##### Reintegration process:

The client’s family is informed and involved in the reintegration process. The CPNs and social welfare officers at the client’s district of residence are engaged to continue community monitoring of the client. Other relevant stakeholders in the community of repatriation engaged before repatriation include: assemblymen, religious leaders, family elders and other relevant opinion leaders in the area of repatriation. The roles of these actors in ensuring the beneficiary stays well are explained to them, and their buy-in is sought. Very importantly, beneficiaries are involved in deciding which of the identified family members they would like to live with and what work or trade they would like to engage in.

##### Post reintegration activities:

The client is linked to a sustainable source of livelihood in his or her community or supported to start a trade. This ensures that the client contributes to his or her upkeep and supports the family.

Social Welfare and CPNs contact clients monthly to check in and provide information on their progress. The monthly follow-up calls are guided by a call guide which collects data such as the beneficiary’s current occupation, whether or not the beneficiary is on medication, how well the beneficiary is doing and any other social or medical challenges the beneficiary is facing. Even though the assessment of how well beneficiaries are doing is not done with a standardised objective tool, the current subjective approach from the perspective of both beneficiaries and a relative provides relevant information that can be compared with subsequent months. The project has considered using a standardised tool to assess how well beneficiaries are doing, but this was not used because of the complexities associated with administering such a long tool on a phone call every month.

## Results

The age of beneficiaries ranged from 21 to 56, with a mean of 36.3 (SD 8.8). Over half of the beneficiaries are females (65.4%). Detailed socio-demographic information of the 81 project beneficiaries as of the end of 2024 can be found in Table 1 below.

**Table 1:** Socio-demographic characteristics of STCF beneficiaries, at the end of 2024.

Background characteristics	Frequency (%)
**Sex**	
Male	28(34.6)
Female	53(65.43)
**Age**	
20-29	19 (23.5%)
30-39	30 (37.0%)
40-49	23 (28.4%)
50-59	9 (11.1)
**Level of Education of Beneficiaries**	
No formal education	21(25.9)
JHS and below	30(37.0)
SHS	18 (22.2)
Diploma/degree	3(3.7)
Masters	1(1.2)
Not known	8(9.9)
**Marital Status of Beneficiaries**	
Single	58(71.6)
Cohabitation	1(1.2)
Married	5(6.2)
Divorced	4(4.9)
Not known	13(16.0)
**Religion**	
Christian	75(92.6)
Muslim	5(6.2)
Traditionalist	1(1.2)

### Employment status prior to ending up on the street

About 59% of beneficiaries were employed in the informal sector prior to ending up on the street. Details of beneficiary employment status prior to ending up on the streets are shown in Table 2 below.

**Table 2:** Employment status prior ending up on the street.

Employment status prior to ending up on the street	Frequency (%)
Unemployed	19 (23.5)
Student	0 (0.0)
informal sector employment	48 (59.3)
Formal sector employment	1 (1.2)
Not known	13 (16.1)

### Duration of stay on the street prior to admission

Most of the beneficiaries had been on the streets for between one to five years. Duration of stay on the street by beneficiaries prior to admission ranged from 11 days to 17 years. Details are shown in Table 3 below.

**Table 3:** Duration of stay on the street prior to admission.

Duration of stay on the street prior to admission	Frequency (%)
Less than 1 year	22 (27.2)
1-5 years	35 (43.2)
Greater than 5 years	2 (2.5)
Not known	22 (27.2)

### Number of children of beneficiaries

The Number of children of beneficiaries ranged from 0 to 7. Details are shown in Table 4 below.

**Table 4:** Number of children of beneficiaries.

Number of children	Frequency (%)
0	28(34.6)
1-3	25 (30.9)
More than 3	4 (4.5)
Not known	24 (29.6)

### History of psychoactive substance use

28.4% of beneficiaries had a history of psychoactive substance use. Details can be found in Table 5 below.

**Table 5:** History of psychoactive substance use.

History of psychoactive substance use	Frequency (%)
Yes	23 (28.4)
No	42 (51.9)
Not known	16 (19.8)

### History of previous psychiatric care

More than half of the project beneficiaries had a history of previous psychiatric care. Details are shown in Table 6 below.

**Table 6:** History of previous psychiatric care.

History of previous psychiatric care	Frequency (%)
Yes	54 (66.7)
No	9 (11.1)
Not known	18 (22.2)

### Registration of beneficiaries on the National Health Insurance Scheme (NHIS)

Just over half of the beneficiaries had been registered on the National Health Insurance Scheme (NHIS) as of 31/12/2024. Details can be found in Table 7 below.

**Table 7:** Registration of beneficiaries on NHIS.

Registration on NHIS	Frequency (%)
Done	43 (53.1)
Not done	38 (46.9)

### Stage of beneficiaries as at the end of 2024(N=81)

The project had repatriated over 60% of its beneficiaries as of 31/12/2024. Details are shown in Table 8 below.

**Table 8:** Stage of beneficiaries as at the end of 2024.

Stage of beneficiaries	Frequency (%)
On admission	24 (29.6)
Repatriated	49 (60.5)
Absconded from the hospital	7 (8.6)
Dead	1 (1.2)

#### Number of beneficiaries enrolled by year.

Since the first beneficiary was enrolled in 2016, 2024 recorded the highest number of new project beneficiaries. Details can be found in Table 9 below.

**Table 9:** Number of beneficiaries enrolled by year.

Year	Number of beneficiaries enrolled (%)
2016	1 (1.2)
2017	3 (3.7)
2018	0 (0)
2019	4(4.9)
2020	6 (7.4)
2021	7 (8.6)
2022	5 (6.2)
2023	24 (29.6)
2024	31 (38.3)

### Diagnosis

Most of the beneficiaries were diagnosed with schizophrenia. Some of the beneficiaries were diagnosed with physical conditions such as conjunctivitis, dyslipidaemia, gestational hypertension, hypertension, CVA, dental caries, cellulitis of a finger, a left shin ulcer and HIV. Details of the various psychiatric diagnoses are shown in Table 10.

**Table 10:** Psychiatric diagnosis of beneficiaries.

Psychiatric diagnosis	Frequency (%)
Schizophrenia	74(91.4)
Substance use disorder	3(3.7)
Schizophrenia and Substance use disorder	2(2.5)
intellectual disability	2(2.5)

### Duration of admission of project beneficiaries

As of the end of 2024, the duration of admission of project beneficiaries ranged from 1 to 71 months, with a mean of nine months for all beneficiaries. Duration of admission for repatriated beneficiaries(N = 49) ranged from 1 month to 41 months with a mean of 6 months and for beneficiaries who were still on admission as of 31/12/2024(N = 24) ranged from 1 to 71 months. Details are shown in Table 11 and Table 12 below.

**Table 11:** Duration of stay for repatriated Beneficiaries.

Duration of stay for repatriated Beneficiaries/ Months	Frequency (%)
1 – 3	27 (55.1)
4- 6	10(20.4)
7-9	7 (14.3)
10 and above	5 (10.2)

**Table 12:** Duration of admission of STCF Beneficiaries still on admission as of 31/12/2024.

Duration of stay for Beneficiaries still on admission/Months	Frequency (%)
1 – 3	6 (25.0)
4- 6	1 (4.2)
7-9	5 (20.8)
10 and above	12 (50)

### Region of repatriation

The majority of repatriated beneficiaries were repatriated to the Greater Accra Region. Four of the beneficiaries were repatriated outside Ghana. Details are shown in Table 13 below.

**Table 13:** Region of Repatriation.

Region of repatriation	Frequency (%)
Greater Accra Region	24
Bono	1
Bono East	1
Central Region	5
Eastern Region	12
Nigeria	1
Sierre Leone	1
Togo	2
Volta	2

### Cost of intervention

The total amount of money spent on the 81 project beneficiaries as at the end of 2024 was Gh₵ 2,835,365.30 (($190,933.69). The total amount spent on the 49 repatriated beneficiaries was GHS1,635,620.00 ($110,143) with an average of GHS33,380 ($2,248). The total cost ranged from GHS 14,019.60 ($944)- GHS 54,743.20 ($3,687).

The UDS rate for December 2024, which was 14.85 cedis to a dollar, was used for the conversions.

The project had spent a total of Gh₵ 2,835,365.30. ($190,933.69) as at the end of 2024. A distribution of costs is shown in Table 14 below.

**Table 14:** Amount spent on intervention as of the end of 2024.

S/N	Category	No. of Beneficiaries	Total cost at the end of 2024/Gh₵	Total cost at the end of 2024/ USD	%
1	Repatriated beneficiaries	49	1,635,620.00	110,143	57.69
2	On admission	40	1,182,800.00	79,650	41.72
3	Deceased	1	16,945.30	1,141	0.60
	Total		2,835,365.30	190,934	100

A total of forty-nine (49) beneficiaries were treated and repatriated under the programme, with treatment period averaging 8 months. The average cost of treatment per beneficiary for the entire process amounted to Gh₵ 33,380.00. A significant portion of this cost, 80.89% (Gh₵ 27,000.00), is attributed to the cost of admission. This suggests that the initial phase of treatment and stabilisation is the most resource-intensive part of the process.

#### Cost distribution.

The average cost of project inputs computed from the 49 repatriated beneficiaries included: pre-pickup surveillance, community engagement and transportation from the streets to Pantang Hospital Gh₵ 1,750.00 constitutes 5.24% of the total cost.

Costs such as hygiene accessories (set of clothing, towel, toothpaste and brush, water bucket, pants, sanitary pad for ladies etc) (Gh₵ 750.00, 2.25%),

Psychiatrist Consultation (Gh₵ 120.00, 0.36%), Routine Laboratory (Gh₵ 450.00, 1.35%), dental care (Gh₵ 310.00, 0.93%), clinical psychology services (Gh₵ 400.00, 1.19%), and medications (Gh₵ 800.00, 2.40%), Occupational Therapy (Gh₵ 1,000.00, 3.00%). Other important components are the cost of medications and follow-up activities after repatriation (Gh₵ 800.00 each, 2.40% each). The cost breakdown is shown in the Table 15 below.

**Table 15:** Table cost components of the STCF Project using the average costs for the 49 beneficiaries who had gone through all stages of the project as at the end of 2024.

S/N	COST BREAKDOWN	AMOUNT/Gh₵	AMOUNT/USD	%
1	Pre-pickup surveillance, community engagement, transportation	1,750.00	118	5.24
2	Psychiatrist Consultation	120.00	8	0.36
3	Hygiene Accessories	750.00	51	2.25
4	Routine Laboratory	450.00	30	1.35
5	Dental care	310.00	21	0.93
6	Clinical psychologist consultation	400.00	27	1.20
7	Occupational Therapy	1,000.00	67	3.00
8	Cost of Follow-up after repatriation	800.00	54	2.40
9	Medications	800.00	54	2.40
10	Cost of admission	27,000.00	1,818	80.89
	**TOTAL COST**	**33,380.00**	**2,248**	**100.00**

### State of beneficiaries on admission (N=24)

Of all the 24 beneficiaries on admission as of 31^st^ December 2024, family tracing for 14 was ongoing, while the families of ten had been successfully traced and contacted. The contacted families were not willing to receive the beneficiaries. The project was engaging relevant stakeholders in their communities to influence such families to accept their relatives.

Four of the beneficiaries on admission were foreign nationals, two from Togo, one from Cote d’Ivoire and one from Benin. The project had written to the embassies of these countries to assist in their repatriation.

Some of the beneficiaries were not well enough to give consistent and reliable information to aid family tracing.

### State of repatriated beneficiaries (N=49)

This section analysed data on 45 instead of the 49 repatriated beneficiaries because four of the foreign nationals repatriated out of Ghana could not be followed up on. Five (11.1%) of the repatriated beneficiaries were engaged in economic activities, while another four had either relapsed or absconded from home. Details of the state of the beneficiaries as of 31/12/2024 are shown in Table 16 below. Specifics on the occupation of beneficiaries are shown in Table 17.

**Table 16:** STCF Beneficiaries state as of 31/12/2024 (N = 45).

Beneficiaries current state	Frequency (%)
Engaged in an economic activity	5 (11.1)
Able to help with household chores but not engaged in any economic activity	25(55.6)
Unable to help with household chores, but does not cause trouble in the community or at home	2(4.4)
Relapsed or absconded from home	4(8.9%)
Could not be reached	9(20.0)

**Table 17:** Occupation of STCF Beneficiaries as of 31/12/2024.

Occupation	Frequency (%)
Teacher	1(2.2)
Aluminium Fabricator	1(2.2)
Security Officer	1(2.2)
Trader	5(11.1)
Unemployed	32(71.1)
Could not be reached	9(20.0)

### Occupation of STCF beneficiaries as of 31/12/2024(N=45)

Over 70% of the repatriated beneficiaries were unemployed. Details are shown in Table 17 below.

### STCF Beneficiaries’ perspective of how well they were doing as of 31/12/2024

About 57% of repatriated project beneficiaries felt they were either well or very well. Details of beneficiaries’ perspective of how well they were doing as of 31/12/2024 are shown in Table 18 below.

**Table 18:** Beneficiaries’ perspective of how well they were doing as of 31/12/2024.

Beneficiaries’ perspective of how well they are doing	Frequency (%)
Very well	10 (22.2)
Well	16(35.6)
Not well	4(8.9)
could not be reached	15(33.3)

### STCF Relatives’ perspective of how beneficiaries were doing as of 31/12/2024

Relatives’ perspective of how beneficiaries were doing as of 31/12/2024 is shown Table 19 below.

**Table 19:** Relatives’ perspective of how beneficiaries were doing as of 31/12/2024.

Relatives’ perspective of how beneficiaries are doing	Frequency (%)
Very well	12(26.7)
Well	14(31.1)
Not well	10(22.2))
could not be reached	9 (20.0)

### Medication intake

An analysis of the proportion of repatriated beneficiaries on medication showed that 53% were on medication. Details are shown in Table 20 below.

**Table 20:** Intake of medications post repatriation.

Intake of medications post repatriation	Frequency (%)
Yes	24(53.3)
No	12(26.7)
Could not be reached	9(20.0)

### Striking quotes from follow-up calls

- *“When I sent her to our district hospital, we were told Health insurance did not cover mental illness hence we were not attended to. So why did you force me to renew her Health insurance?”* **a relative of a STCF beneficiary speaking in an angry tone**
- “*His family says he is cursed and must stay* *mad, hence they do not allow me to give him his medication”* **a CPN in the VR**
- ***“*** *My new workplace said they will not allow me to leave work and come for review”* ***a* STCF Beneficiary**
- ***“*** *I am the only one at home and I have to go to work, so I can’t bring her for review*” **a relative of a STCF Beneficiary**

### Key challenges facing the project

Some challenges facing the project include a sedentary lifestyle of beneficiaries while on admission due to the limited physical activity of clients on the ward. Inadequate staff strength at the facility level, as well as inadequate CPN at the district level, are also challenges faced by the project. There has been a difficulty in bringing all identified stakeholders on board to buy into the intervention. Another challenge is the low mental health literacy in Ghanaian communities.

The intervention also faces a difficulty in tracing the families of beneficiaries. In addition, traced family members are sometimes reluctant to receive their relatives when beneficiaries are well enough to be seen on OPD basis.

Lack of cooperation from some District Assemblies and embassies is also a challenge faced by the project.

Finally, the project lacks a van and motorcycles to aid pre-pick up surveillance, home tracing and home visits to repatriated beneficiaries.

### Innovative strategies adopted by the project

- Monthly follow-up calls
- Sending medications via public transport to beneficiaries
- Transportation allowance to support beneficiaries who come to Pantang Hospital for review (given to only those who need it based on the assessment of the social workers)
- Occasional follow-up visits to beneficiaries in Accra
- Use of social media to aid family tracing
- Fundraising in churches

## Discussion

The STCF project seeks to give beneficiaries a second chance at working productively and contributing to the development of society. In enrolling beneficiaries, priority is given to younger persons because they have more productive years ahead of them. This explains why most of the beneficiaries were around age 30. There were instances where sponsors decided exactly who they wanted enrolled, not paying attention to the age of the person, which explains why some beneficiaries were above the age of 40 years at enrolment.

Considering most of the beneficiaries, aged between 21 and 56 years, had been diagnosed with schizophrenia, this is in line with established literature revealing the age range for onset of schizophrenic illness, mostly, to be between 15–54 years. It buttresses Professor Nancy Andreasen’s declaration that the illness afflicts a lot of people found on the streets [26,27], as well as supporting studies that have discerned high prevalence of psychiatric conditions amongst homeless populations [8–16,19–22]. It must be noted that the higher prevalence of psychiatric conditions in this intervention population may be explained by the methodology of fetching suspected mentally ill homeless persons.

In the realm of educational achievements, 85.1% were unable to attain tertiary education, much like 83.4% from Nwaopara and associates [20]. This could be attributable to, presumably, the onset of illness during the age frame for achievement of such educational milestones. The current literacy rate of Ghana stands at 76.6% [28], with that of beneficiaries at 64.1% having some form of formal education.

Sufficient social support is an integral part of the management of mental disorders as well as outcomes. Marriage has been investigated and found to be prognostically positive for people with mental disorders [29]. It was thus not surprising to find most of the beneficiaries were single, with a few divorced.

This finding was consistent with those in a sister intervention from two countries eastward [19]. The classic chicken-egg conundrum is encountered here with failure to determine if mental illness contributes to being single, with the additional possibility of dissolution of relationships due to illness, or if being single contributes to the development of mental illness.

It also does not come as a surprise that most of the beneficiaries were Christian; this mirrors the nationwide estimate of 71.3% as of 2021 [28–30].

In Ghana, 80–95% of employment stems from the informal sector [31,32], therefore, informal employment dominating amongst beneficiaries was expected. 23.5% were unemployed prior to illness onset – this reflects employment troubles amongst mentally ill populations [33]. This highlights the need for policies and implementation of existing policies that ensure more job opportunities and a convenient work environment for persons living with mental illness.

About four out of every ten beneficiaries had spent between a year and five years on the streets before being picked up. Duration of stay on the street may be proxied as duration of illness as well as duration of neglect by social support systems, thereby emphasising the relevance of George Engel’s biopsychosocial model [34]. This finding stresses the need for more interventions that target this vulnerable population. It also calls on the government of Ghana to allocate more resources to research and initiatives that focus on this group. Also, seeing such a visible vulnerable population neglected for this long raises questions about the weight the relevant stakeholders place on mental health.

A little over half of the beneficiaries reported no history of substance use, consistent with established literature elaborating the interplay between schizophrenic illness and substance use [35]. This finding also emphasises the significant contribution of illicit substance use to this public health problem, hence the need for key actors to intensify the fight against illicit substance use.

Almost seven out of every ten beneficiaries had a history of contact with mental health services but were lost to follow-up. This could emphasise the strain of mental health disorders on individuals, families and larger societies, considering significant resources involved in their management [6,36–39]. Could this be a commendation for the Ghanaian mental health framework? Considering a previous mental service contact rate of 10% for homeless people with psychosis in Ethiopia [21]. This finding also raises the question of the factors that could have contributed to individuals with previous psychiatric care ending up on the street, an area for future study.

This intervention seeks to be a vehicle for universal health coverage (UHC), ensuring access to both mental and physical health services irrespective of ability to pay at the point of use. To achieve this goal without overburdening the limited financial resources available to the project, the project tries to enrol all beneficiaries on the national health insurance scheme (NHIS). Even though the NHIS does not cover mental health services, the cost of the general health needs of beneficiaries will be covered. As of December 2024, just a little over half of beneficiaries had been enrolled on the NHIS, even though registration is free for this vulnerable population. The project invites the NHIS office close to the hospital routinely to register and renew the NHIS of project beneficiaries, staff and the surrounding community. Beneficiaries who are enrolled and repatriated before an NHIS registration exercise are encouraged to do so on their own at repatriation and during the monthly follow-up calls.

This raises concerns about other possible bottlenecks to enrolling beneficiaries on the NHIS, other than registration fees.

The low number of beneficiaries since the onset of the project is due to limited funds. The project is putting in efforts to reach more prospective sponsors to help overcome this bottleneck. The disparity in the number of beneficiaries enrolled in the different years has been mainly due to the funds available to the hospital and the other competing needs of the hospital in those years.

The mean duration of stay of both reintegrated and beneficiaries still on admission of nine months was longer than the expected three-month duration that was used for planning. This has mainly been due to the challenges associated with stabilisation of maladies, tracing families and the reluctance of some traced family members to accept the beneficiary. The project hopes to overcome the challenge of tracing family members by partnering with the Ghana Police Service and the media houses to display beneficiaries as missing but found. The project has also used Facebook to successfully trace one of the project beneficiaries who was a foreign national. Reluctance of traced families to accept beneficiaries could be due to past unpleasant experiences of living with such beneficiaries. One beneficiary who had attempted to kill all the different relatives he had ever lived with was described by an uncle as “a devil” left behind by his dead parents. This observation could also be from the stigma associated with having a relative with mental illness. When the beneficiaries are successfully linked to sustainable sources of livelihood, such that they will not be a burden on their families, family members may be more likely to receive them. This will be aided by the mental health education that family members receive from the project.

Some of the sponsors provided start-up capital for the beneficiaries they sponsored. This has helped to compensate for the void created by the challenge of linking beneficiaries to existing social intervention schemes.

Some families readily accepted their relatives who had benefited from the project when the project assured them of monthly support funds. Unfortunately, the project was unable to honour this promise because the prospective partner who pledged this support did not deliver. The duration of stay at the hospital could be reduced in the presence of reliable support for beneficiaries after reintegration into the community.

The project repatriates beneficiaries to the homes of traced families who are willing to receive them, be it in Ghana or outside Ghana.

Repatriating beneficiaries across Ghana and three different African countries suggests that the STCF Project, if well established and funded, will not only benefit Ghanaians but also foreign nationals.

The most significant predictor of project cost is the cost of admission, which correlates with the duration of stay at the hospital. The longer the duration of stay of project beneficiaries, the higher the cost of the intervention.

Long-term follow-up of reintegrated beneficiaries is a vital aspect of the intervention. The goal is to reduce the proportion of beneficiaries who end up on the streets again. To ensure sustainability, the project employs the existing district community psychiatric network and the district social welfare structure. The monthly follow-up calls and support from the project team at Pantang Hospital provide a post-reintegration surveillance system which monitors beneficiary wellbeing, intake of medication, attendance for reviews and engagement in economic activities, among others. A relapse of almost nine out of every ten repatriated project beneficiaries shows the complex nature of the problem and calls for more research to better understand and address this challenge.

The striking quotes extracted from the monthly follow-up call records of CPNs and social welfare officers raise major concerns worthy of discussion at the national agenda table.

The NHIS, the country’s main vehicle for UHC, does not cover mental health care. Even though the Ministry of Health and the National Health Insurance Authority have promised coverage for selected mental health conditions, the proposal does not cover inpatient care and services received at the tertiary psychiatric hospitals. This makes out-of-pocket payment the main approach for financing Mental Health care in Ghana, an occurrence that could lead to catastrophic health expenditure.

The second quote throws light on the impact of some bad traditional beliefs on access to mental health care and long-term treatment outcomes. A cultural belief that prevents the health system from sending needed mental health services to the mentally ill at their homes is not likely to promote sending such patients to the hospital for their planned reviews. An occurrence that draws back the long-term success of this intervention. There is therefore a need for continuous community stakeholder engagements that will target improving mental health literacy and debunking cultural beliefs and practices that negatively impact mental health.

To ensure long-term treatment success, there is a need for responsive workplace mental health policies that will provide a friendly and supportive work environment for persons living with mental illness. In the case of this beneficiary, the project team considered a visit to his workplace to engage his employers, but the dilemma that held us back was whether it was ethical to disclose the client’s mental health status to his employers.

The last of the striking quotes reported in this paper brings to light the challenges relatives face when they have to make subtle to significant adjustments to their daily life routines to accommodate relatives who have been out of their lives for years. Even though this particular relative only commented on the difficulty associated with making time to bring the beneficiary for his reviews, other possible adjustments required are preparing accommodation units for beneficiaries and financial allocation to feed and clothe an extra person. Responsible governmental and non-governmental organisations need to intensify support to persons with mental illness and their support systems.

There is a need to continuously collect and analyse project data to inform project implementation and to constantly explore innovative solutions to implementation bottlenecks.

### Limitations of the intervention

The project had hoped that both government and non-governmental organisations would provide support for various phases of the intervention, but this has not been the case. Repatriated beneficiaries have not received the anticipated skill training, job opportunities and financial support for their support systems.

The use of a simple subjective tool to assess how well beneficiaries are doing post-repatriation.

Beneficiaries whose families are not identified remain on admission at the hospital even when they are well enough to be seen on an OPD basis. This reduces the number of empty beds for new clients. The project has no sustainable source of funding. Efforts to engage all the stakeholders relevant to this project had not been successful as of December 2024.

### Limitations of this study

There were some data that could not be assessed because some beneficiaries were not well enough to provide the information. The costing of the intervention could have been more detailed to include indirect as well as intangible costs. There is a need to conduct a detailed economic evaluation of this intervention.

There are a lot of ethical dilemmas faced by this intervention, some of which include:

1. In the face of limited resources available to the intervention, how is one mentally ill person on the street selected over the other (rationing of scarce resources)
2. Publicising the pictures of project beneficiaries using conventional and social media to help trace their families
3. The ethics of the forceful pick up of beneficiaries from the streets using physical or chemical restraint
4. The ethics of showing before and after pictures of project beneficiaries to help raise funds or communicate project impact
5. The ethics surrounding how to manage beneficiaries whose traced families do not want to receive them, or whose families are not traced after a long period
6. The ethics of reviewing before and after pictures with beneficiaries to improve insight and treatment adherence.
7. The ethics of involving relevant community opinion leaders during the repatriation process (this involves educating them on the beneficiaries’ diagnosis and the supportive roles they need to play to reduce the chances of relapse).

Impediments in the domain of resources are similar to those ascertained in a study pertaining to low-and middle-income countries by Giebel et al. [40].

Most of these have been considered but not piloted because of the associated ethical complexities.

There is a pressing need for a separate study that will focus on these ethical dilemmas.

### Conclusion and recommendation

The “Set the Captives Free” project, if given the needed sponsorship and support from all the necessary stakeholders, can become the model approach for addressing the public health problem of homeless mentally ill persons living on the streets. Active participation and buy-in of all stakeholders are key to the success of the project. This paper has raised more questions than answers, stressing the need for more research in the areas of ethics, health economics and cultural beliefs relevant to addressing this public health concern.

The STCF approach emphasises the use of existing national structures to address this challenge, making the intervention sustainable. The MOH should consider setting up a national programme similar to the Tuberculosis Control or Malaria Control programmes to scale up this intervention across the country.

## Supporting information

S1 Text Data compailation form. (DOCX)

S2 Text Follow- up call guide. (DOCX)

S1 Data Deidentified data. (CSV)

S2 Data Deidentified follow-up calls data. (XLSX)

S3 Data Project financial data. (XLSX)

## References

[1] Abekah-Carter K, Oti GO. Perspectives of community members on homeless people with mental illness in Nsawam, Ghana. Int J Soc Psychiatry. 2022;68(1):196–202. doi: 10.1177/0020764020984195 33356747

[2] Prince M, Patel V, Saxena S, Maj M, Maselko J, Phillips MR, et al. No health without mental health. Lancet. 2007;370(9590):859–77. doi: 10.1016/S0140-6736(07)61238-0 17804063

[3] Republic of Ghana. Mental Health Act. Act 846. Accra: Ghana Publishing Company; 2012.

[4] Adu-Gyamfi S. Mental Health Service in Ghana: A Review of the Case. IJPHS. 2017;6(4):299. doi: 10.11591/ijphs.v6i4.8474

[5] Walker GH, Osei A. Mental health law in Ghana. BJPsych Int. 2017;14(2):38–9. doi: 10.1192/s2056474000001768 29093937 PMC5618813

[6] Addo R, Nonvignon J, Aikins M. Household costs of mental health care in Ghana. J Ment Health Policy Econ. 2013;16(4):151–9. 24526584

[7] Mfoafo-M’Carthy M, Sossou MA. Stigma, discrimination, and social exclusion of the mentally ill: the case of Ghana. Journal of Human Rights and Social Work. 2017;2(4):128–33. doi: 10.1007/s41134-017-0043-2

[8] Fazel S, Khosla V, Doll H, Geddes J. The prevalence of mental disorders among the homeless in western countries: systematic review and meta-regression analysis. PLoS Med. 2008;5(12):e225. doi: 10.1371/journal.pmed.0050225 19053169 PMC2592351

[9] Nielsen SF, Hjorthøj CR, Erlangsen A, Nordentoft M. Psychiatric disorders and mortality among people in homeless shelters in Denmark: a nationwide register-based cohort study. Lancet. 2011;377(9784):2205–14. doi: 10.1016/S0140-6736(11)60747-2 21676456

[10] Perry J, Craig TKJ. Homelessness and mental health. Trends Urol Mens Health. 2015;6(2):19–21. doi: 10.1002/tre.445

[11] Bhui K, Shanahan L, Harding G. Homelessness and mental illness: a literature review and a qualitative study of perceptions of the adequacy of care. Int J Soc Psychiatry. 2006;52(2):152–65. doi: 10.1177/0020764006062096 16615247

[12] Tinland A, et al. Psychiatric disorders and addiction among homeless people in Marseille, France: evaluating integrated housing and care programs. BMC Psychiatry. 2018;18(1):314.30261864

[13] Stergiopoulos 1 3 V, Herrmann C, Rourke P, Dunn SJ, Hwang SW. Mental health, service use, and barriers to care among homeless individuals in Toronto. Can J Psychiatry. 2015;60(10):459–65.

[14] Chamberlain C, Johnson G. Pathways into adult homelessness. J Sociol. 2011;49(1):60–77. doi: 10.1177/1440783311422458

[15] Kudo T, Fukui N, Imai S. Mental disorders among homeless people in Tokyo: Prevalence and associated factors. Psychiatry Clin Neurosci. 2020;74(5):273–81.

[16] Wong YC, Lee C, Tang K. Homelessness and mental health among marginalized populations in Hong Kong: A qualitative study. Asia Pac J Soc Work Dev. 2021;31(1–2):70–84.

[17] United Nations. The Sustainable Development Goals Report. United Nations; 2020. doi: 10.18356/2282dd98-en

[18] Frye EA, McQuistion HL. Psychiatry in the streets: Unique services for people experiencing homelessness. Psychiatric Times. 2016;33(7).

[19] Fekadu A, Hanlon C, Gebre-Eyesus E, Agedew M, Solomon H, Teferra S, et al. Burden of mental disorders and unmet needs among street homeless people in Addis Ababa, Ethiopia. BMC Med. 2014;12:138. doi: 10.1186/s12916-014-0138-x 25139042 PMC4147171

[20] Nwaopara 20 U, Abasiubong F, Umoh O. Mental health care of homeless mentally ill patients in Akwa Ibom state, Nigeria: rehabilitation model, challenges and strategies for improvement. J Int Res Med Pharmaceut Sci. 2016;10(2):60–8.

[21] Hossain MM, Sultana A, Tasnim S, Fan Q, Ma P, McKyer ELJ, et al. Prevalence of mental disorders among people who are homeless: An umbrella review. Int J Soc Psychiatry. 2020;66(6):528–41. doi: 10.1177/0020764020924689 32460590

[22] Hwang SW, Weaver J, Aubry T, Hoch JS. Hospitalization and ambulatory care use among homeless adults: a population-based study. Medical Care. 2011;49(4):350–4. doi: 10.1097/MLR.0b013e318206c50d 21368678

[23] Poulin S. Victimization of Mentally Ill - Mental Illness Policy Org. Mental Illness Policy Org. [cited 2021 Oct 27]. Available from: https://mentalillnesspolicy.org/consequences/cost-homeless-mentally-ill.html

[24] Ghana Statistical Service. 2021 Population and Housing Census - Ghana Statistical Service: General report on population and housing census 2021. Ghana Statistical Service; 2021 [cited 2023 Mar 16]. [Online]. Available from: https://census2021.statsghana.gov.gh/bannerpage.php?readmorenews=MTM3MjM1Mzg1OS41Nzg=&amp;Release-of-Provisional-Results

[25] Bone TA, Roberts M. An investigation into the routes to inpatient care at the Pantang Hospital in Ghana via the criminal justice system. Ghana Med J. 2019;53(2):100–8. doi: 10.4314/gmj.v53i2.4 31481805 PMC6697773

[26] Jablensky A. Schizophrenia and psychotic disorders. In: Geddes JR, Andreasen NC, Goodwin GM, editors. New Oxford Textbook of Psychiatry. 3rd edition. Oxford: Oxford University Press. 2020. p. 568–78.

[27] Geddes JR, Andreasen NC, Goodwin GM. Schizophrenia and psychotic disorders. New Oxford Textbook of Psychiatry. 3rd edition. Oxford: Oxford University Press; 2020. p. 568–78.

[28] Williams K, Frech A, Carlson DL. Work and mental health. In: Scheid TL, Brown TN, editors. A Handbook for the Study of Mental Health. 2nd edition. New York: Cambridge University Press; 2010. p. 306–20.

[29] United Nations Department of Economic and Social Affairs, Population Division. Population of Ghana. United Nations. [cited 2025 Apr 18]. Available from: https://countrymeters.info/en/Ghana

[30] Ghana Statistical Service. 2021 PHC General Report Vol. 3C: Background Characteristics. Ghana Statistical Service. 2021. [Online]. Available from: https://census2021.statsghana.gov.gh/gssmain/fileUpload/reportthemelist/2021%20PHC%20General%20Report%20Vol%203C_Background%20Characteristics_181121.pdf

[31] Informal sector makes up 80% of Ghana’s workforce; its contribution to GDP only 27.4%. MyJoyOnline. [cited 2026 Jan 31]. Available from:https://www.myjoyonline.com/informal-sector-makes-up-80-of-ghanas-workforce-its-contribution-to-gdp-only-27-4/

[32] Baah-Boateng W, Vanek J. Informal workers in Ghana: A statistical snapshot. WIEGO Statistical Brief No. 21, Women in Informal Employment: Globalizing and Organizing. 2020. Available from: https://www.wiego.org/wp-content/uploads/2020/03/WIEGO_Statistical_Brief_N21_0.pdf

[33] Luciano A, Meara E. Employment status of people with mental illness: national survey data from 2009 and 2010. Psychiatr Serv. 2014;65(10):1201–9. doi: 10.1176/appi.ps.201300335 24933361 PMC4182106

[34] Engel GL. The need for a new medical model: a challenge for biomedicine. Science. 1977;196(4286):129–36. doi: 10.1126/science.847460 847460

[35] Khokhar JY, Dwiel LL, Henricks AM, Doucette WT, Green AI. The link between schizophrenia and substance use disorder: a unifying hypothesis. Schizophr Res. 2018;194:78–85. doi: 10.1016/j.schres.2017.04.016 28416205 PMC6094954

[36] Knapp M, Iemmi V. Mental health. In: Scheffler RM, editor. World Scientific Handbook of Global Health Economics and Public Policy. Singapore: World Scientific; 2016. p. 1–41.

[37] Trautmann S, Rehm J, Wittchen H-U. The economic costs of mental disorders: Do our societies react appropriately to the burden of mental disorders? EMBO Rep. 2016;17(9):1245–9. doi: 10.15252/embr.201642951 27491723 PMC5007565

[38] World Economic Forum. The global economic burden of non-communicable diseases. World Economic Forum; 2011. Available from: www.weforum.org/EconomicsOfNCD

[39] Arias D, Saxena S, Verguet S. Quantifying the global burden of mental disorders and their economic value. eClinicalMedicine. 2022;54:101675. doi: 10.1016/j.eclinm.2022.101675 36193171 PMC9526145

[40] Giebel C, Gabbay M, Shrestha N, Saldarriaga G, Reilly S, White R, et al. Community-based mental health interventions in low- and middle-income countries: a qualitative study with international experts. Int J Equity Health. 2024;23(1):19. doi: 10.1186/s12939-024-02106-6 38308294 PMC10835969

