# Supplementary material for: Integrating homeless persons with mental health conditions back into low resource communities: A cross-sectional study
Source: PLOS Ment Health. 2026 Mar 20;3(3):e0000510. doi: 10.1371/journal.pmen.0000510 (PMC13004371; doi:10.1371/journal.pmen.0000510)
Supplement: S1 Text — (DOCX) [file pmen.0000510.s001.docx]

**Set the Captives Free data compilation form**

**Part 1 : DEMOGRAPHICS**

1. Sex… 0- male 1- Female
2. Age……………
3. Marital status: 0- Single 1- Cohabitation 2- Married 3- Divorced 4-Widowed
4. Religion: 0- Christian 1- Muslim 2- Traditionalist 3- Other ……………………….
5. Level of education Completed: 0- no formal education 1-JHS and below 2- SHS 3- Diploma/degree 4- Masters and above
6. Employment status prior to admission. 0- Unemployed 1- student 2- informal sector employment 3- formal sector employment, specify
7. Previous psychiatric treatment received prior to ending up on the street. 0- none 1- Medical 2- faith based treatment 3- herbal
8. Number of children…..
9. Duration of stay on the street prior to admission (in years, please use 99 for less than 1 year )…………..
10. Date of pick up ….
11. District of pick up…………
12. Still on admission … 0- Yes 1- No 2-Absconded
13. History of psychoactive substance use… 0- yes 1- no
14. Primary Sponsor…… 0- Pantang Hospital 1- Religious body 2- cooperate body 3- individuals
15. Psychiatric diagnosis… 0- Schizophrenia 1- Substance use disorder 2- intellectual disability 3- BAD
16. Link to sustainable source of livelihood….. 0- yes 1- no
17. NHIS status 0- done 1- not done
18. Family tracing --- 0- successful 1- not successful
    - - 1. Medical diagnosis
        2. Psychiatric diagnosis
        3. Cost of care
        4. Date of repatriation
        5. District of repatriation
        6. Duration of stay
        7. Region of repatriation

***NB Kindly Use CODE 9 for inaccessible information***
