# Supplementary material for: Integrating homeless persons with mental health conditions back into low resource communities: A cross-sectional study
Source: PLOS Ment Health. 2026 Mar 20;3(3):e0000510. doi: 10.1371/journal.pmen.0000510 (PMC13004371; doi:10.1371/journal.pmen.0000510)
Supplement: S2 Text — (DOCX) [file pmen.0000510.s002.docx]

**STCF FOLLOW UP CALLS INTERVIEW GUIDE**

1. Date of follow up call………………………..
2. Name of staff calling………………………………………………
3. Name of client called…………………………………
4. Time of call…………………
5. Beneficiary’s current activity 0- Engaged in an economic activity 1- able to help with household chores but not engaged in any economic activity 2- unable to help with household chores but does not cause trouble in the community or at home 3- Relapsed or absconded from home
6. Beneficiary’s current occupation…………..
7. How well is beneficiary doing (beneficiaries perspective)? 0- Very well 1- well 2- not well
8. How well is beneficiary doing (3^rd^ party’s perspective)? 0- Very well 1- well 2- not well
9. Is client taking his/her medication? 0- Yes 1- No
10. Is Beneficiary experiencing any social or medical challenges? 0- Yes 1- No. If no skip to question 12
11. Summary of beneficiaries challenges ……………………………………………………………………………………………..……………………………………………………………………………………………..
12. Date of last review…………………..
13. Date for next review ………………….
14. Date for next follow up call………………….
